# Competing effects of spreading rate, crystal fractionation and source variability on Fe isotope systematics in mid-ocean ridge lavas

**DOI:** 10.1038/s41598-021-83387-7

**Published:** 2021-02-18

**Authors:** Marianne Richter, Oliver Nebel, Martin Schwindinger, Yona Nebel-Jacobsen, Henry J. B. Dick

**Affiliations:** 1 Isotopia Laboratory, School of Earth, Atmosphere and Environment, Monash University, Clayton, VIC 3800 Australia; 2 Department of Geology and Geophysics, Woods Hole Oceanographic Institution, Woods Hole, MA 02543-1539 USA

**Keywords:** Geochemistry, Geology, Petrology, Volcanology

## Abstract

Two-thirds of the Earth is covered by mid-ocean ridge basalts, which form along a network of divergent plate margins. Basalts along these margins display a chemical diversity, which is consequent to a complex interplay of partial mantle melting in the upper mantle and magmatic differentiation processes in lower crustal levels. Igneous differentiation (crystal fractionation, partial melting) and source heterogeneity, in general, are key drivers creating variable chemistry in mid-ocean ridge basalts. This variability is reflected in iron isotope systematics (expressed as δ^57^Fe), showing a total range of 0.2 ‰ from δ^57^Fe =  + 0.05 to + 0.25 ‰. Respective contributions of source heterogeneity and magma differentiation leading to this diversity, however, remain elusive. This study investigates the iron isotope systematics in basalts from the ultraslow spreading Gakkel Ridge in the Arctic Ocean and compares them to existing data from the fast spreading East Pacific Rise ridge. Results indicate that Gakkel lavas are driven to heavier iron isotope compositions through partial melting processes, whereas effects of igneous differentiation are minor. This is in stark contrast to fast spreading ridges showing reversed effects of near negligible partial melting effects followed by large isotope fractionation along the liquid line of descent. Gakkel lavas further reveal mantle heterogeneity that is superimposed on the igneous differentiation effects, showing that upper mantle Fe isotope heterogeneity can be transmitted into erupting basalts in the absence of homogenisation processes in sub-oceanic magma chambers.

## Introduction

Mid-ocean spreading ridges extend for more than ~ 60,000 km on Earth and show a wide range in spreading rate. The spreading rate varies between 6 and 150 mm per annum (mm/a), in the Arctic and Pacific Oceans, respectively^1,2^. These large variations in spreading rate can be linked to differences in the styles of partial melting regimes^1,3–9^, which in turn is affected by differences in mantle potential temperatures, rift geometry and associated kinematics^1,4,10–12^. Based on those characteristics four distinct spreading regimes can be classified: ultraslow (< 14 mm/a), slow (14–55 mm/a), intermediate (55 – 80 mm/a) and fast spreading ridges (> 80 mm/a)^1,13,14^. Each spreading regime has its own characteristics contributing to the chemical diversity and complex nature of mid-ocean ridge basalts^15,16^.

For instance, the mantle beneath ultraslow (< 14 mm/a) spreading ridges is colder, promoting rapid melt transport through dikes from weak and small mantle upwelling cells into crustal levels^3,14^. Hence, during melt ascent, melt is not channelled through sub-oceanic magma lenses (or ‘chambers’), in which melt potentially undergoes replenishment and fractional crystallisation, as proposed for many faster ridges worldwide^9^. At intermediate and fast spreading ridges (> 55 mm/a) melt migrates in incremental steps from the source region towards the surface, in which the melt undergoes geochemically changes due to fractional crystallisation, melt replenishment in axial magma chambers, magma mixing and melt-rock interaction^6,17,18^.

Inevitably, the remote nature of sub-volcanic processes poses a problem in identifying processes leading to volcanic eruption products. A better understanding of these processes, i.e., melt migration, melt-rock reaction and fractional crystallization processes can be gained by petrographic studies of lower oceanic crust rocks^17,19^. This can be often complemented by studies of xenocrysts in ocean floor lavas as well as experimental models^20–23^. An additional tool to understand the magmatic evolution at mid-ocean ridges are stable isotope systematics^24–31^. These provide further insights into magmatic differentiation processes due to kinematic isotope fractionation, which can further translate into understanding thermal differences at ocean ridges (i.e., spreading rate effects, partial melting and crystal fractionation).

Melt at oceanic spreading ridges originate from an ultramafic source (mantle peridotite or mantle pyroxenite) and fractionates during the ascent from the upper mantle *en route* to surface to a more mafic melt composition (gabbro/basalt)^32^. During the fractionation process ultramafic Fe–Mg-rich minerals (olivine, pyroxene) are removed from the melt, thus creating a change in geochemistry of the melt *en route* to the surface^33^. Iron is a major element in most mantle minerals, including olivine and pyroxene, and occurs as ferric (Fe^3+^) and ferrous (Fe^2+^) species in the *f*O_2_ range of the upper mantle and the crust^34,35^. Each iron species requires a different bonding environment between crystallizing phases and basaltic melt, resulting in mass-dependent isotope fractionation during crystal fractionation^36^. Valence bond theory predicts that ferrous iron is preferentially associated with lighter iron isotopes^37^, whereas ferric iron may subsequently be associated with heavier iron isotopes (expressed here as δ^57^Fe relative to IRMM-524A)^38^. Iron isotope studies of mid-ocean ridge basalts (MORBs) showed that source heterogeneity^39^ and magmatic differentiation processes effect δ^57^Fe^40–48^. Olivine plays a critical role during magmatic differentiation processes, because when removed from the melt during crystal fractionation, it is expected to cause a shift in δ^57^Fe along the liquid line of descent. This is convincingly illustrated in mid-ocean ridge basalts from fast spreading ridges^40,49^. It is, however, unclear to which extend, both, source heterogeneity and igneous differentiation, effect iron isotope composition, and if this can be linked to a difference in spreading rate.

To investigate this, we studied the Fe isotope composition of lavas from the Gakkel Ridge in the Arctic Ocean. The Arctic ridge system is classified as an ultraslow spreading ridge^1^ due to its variable magmatic activity in relation to spreading rate, which ranges from 15 mm/a in the West to 6 mm/a in the East,^1,50^. Only a few studies on Fe isotope systematics in MORBs sampled basalts from a variety of oceanic ridge locations ranging from fast to intermediate to slow spreading rates^25,28,56^. Results of these studies show that δ^57^Fe in MORBs range between + 0.07 and + 0.22‰ for variable MgO of 4.7–8.5 wt%, which is plausibly linked to magmatic differentiation processes. To the best of our knowledge, however, no study to date has investigated the iron isotope systematics in ocean ridge basalts from ridges with ultraslow spreading rates < 13 mm/a, where magmatic differentiation is expected to be limited compared to those from fast spreading ridges. Gakkel Ridge lavas further record heterogeneous mantle sources^51–55^, which can add variability on Fe isotope systematics. Crystal fractionation is not the only driver that affects the composition of the melt during the ascent from the parental source to the surface, and ultimately the Fe isotope composition. Mantle source heterogeneity also contributes to a chemical diversity in oceanic ridge basalts. The Arctic ocean ridge possesses the unique character of extreme mantle heterogeneity, ranging from an enriched mantle in the West^51,53^ to highly depleted ridge sections in the East^54,55^. This is ideal to not only study if magmatic differentiation is a key factor for iron isotope variability in mid-ocean ridge basalts, but also to understand the role of mantle heterogeneity for iron isotope signatures in basaltic melts. In this contribution, we present new iron isotope data from the ultraslow Arctic mid-ocean ridge and compare these results to existing data from faster spreading regimes. This comparison allows an assessment of both, effects of spreading rates and source variability, on Fe isotopes in mid-ocean ridge lavas.

## Results

**Figure 1:**
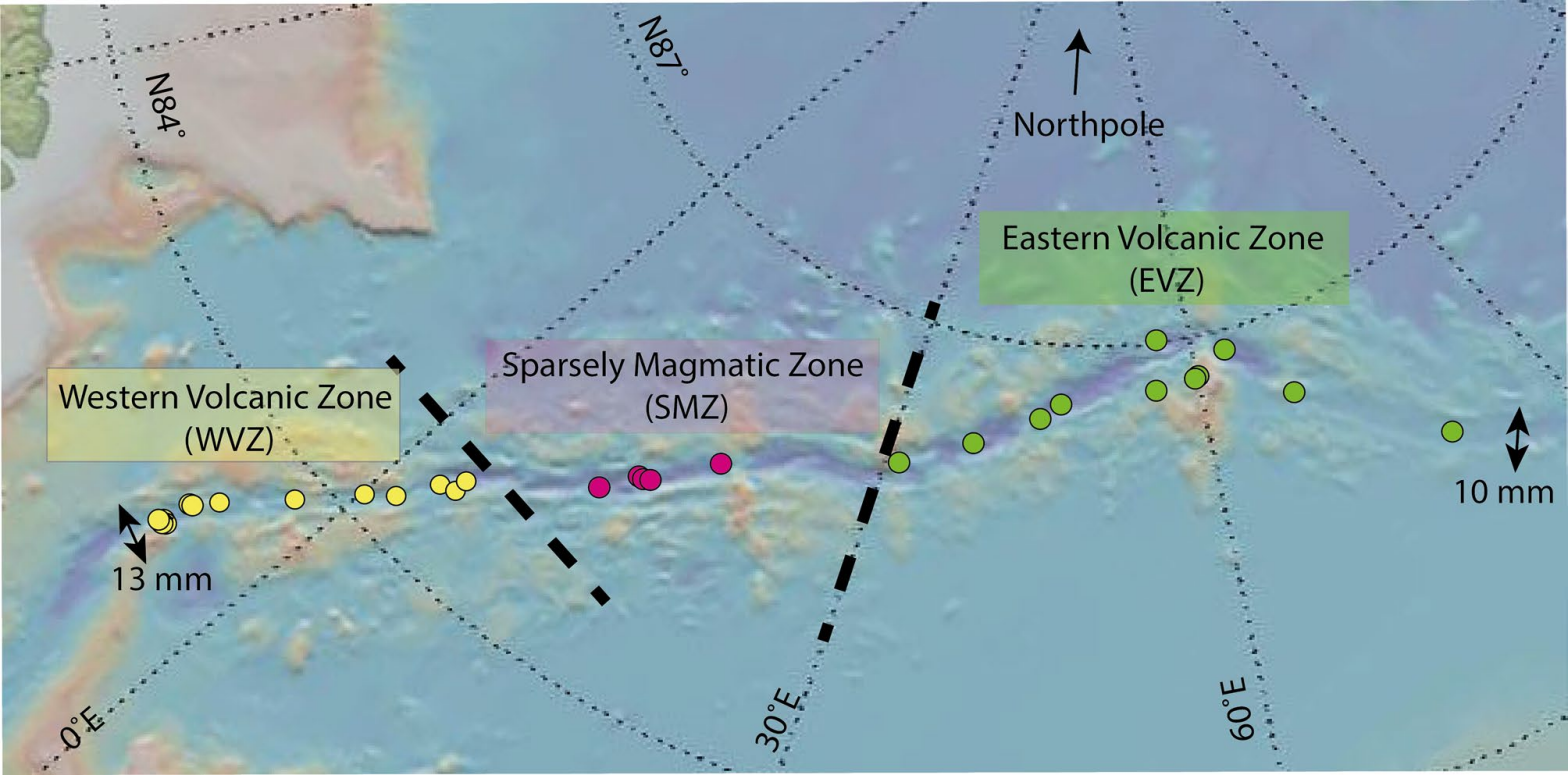
Sample locations. The Gakkel Ridge extends for 1800 km from the Northeast of Greenland to the Northwest of Siberia and can be divided into three different ridge segments—the Western Volcanic Zone (WVZ), the Sparsely Magmatic Zone (SMZ) and the Eastern Volcanic zone (EVZ)^50^. All three segments are highlighted in a different colour (yellow = WVZ; pink = SMZ; light green = EVZ) and are separated by a black dashed line that indicates the local extension of each ridge segment, based on petrological and tectonic observations^50^. The map was made using GeoMapApp (http://www.geomapapp.org).

**Figure 2:**
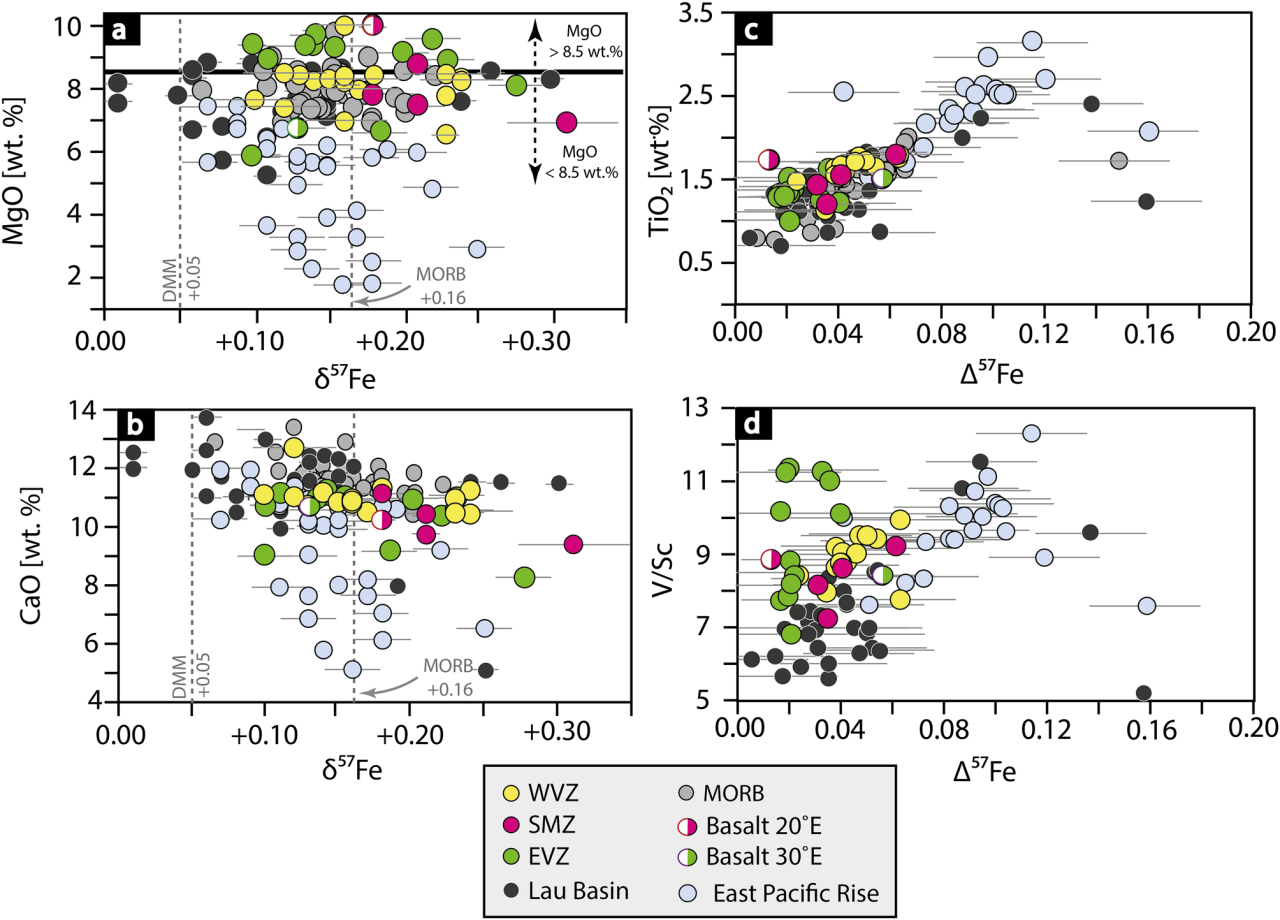
Major and trace elements versus iron isotope systematics. **(a)** MgO versus δ^57^Fe shows no obvious correlation. The black bold line marks the difference between the primitive (> 8.5 wt% MgO) and more evolved (< 8.5 wt% MgO) Gakkel basalts. **(b)** CaO versus δ^57^Fe. Gakkel Ridge lavas have lower CaO values compared to MORBs globally. **(c)** TiO_2_ versus Δ^57^Fe (Δ^57^Fe = δ^57^Fe − δ^57^Fe_prim_; see text for detailed description). Δ^57^Fe is used here as an indicator for crystal fractionation with larger values marking higher degrees of fractionation. The major element values show a positive correlation with increasing isotope fractionation (for Δ^57^Fe < 0.12 ‰). Advanced isotope fractionation (Δ^57^Fe > 0.12 ‰) shows a negative correlation with TiO_2_. **(d)** V/Sc versus Δ^57^Fe shows similar behaviour with increasing isotopic fractionation as TiO_2_. Basalts from the EVZ, however, do not follow this trend and scatter at around Δ^57^Fe = 0.02–0.03 ‰. MORB data are compiled from Teng et al.^25^, Weyer et al.^45^ and Chen et al.^49^; East Pacific Rise data from Chen et al.^48^, and data from the Lau Basin from Nebel et al.^40^ are used here for comparison.

Thirty-five dredged, fresh basalts from three different tectonomagmatic segments, the Western Volcanic Zone (WVZ), the Sparsely Magmatic Zone (SMZ) and the Eastern Volcanic Zone (EVZ) from the Gakkel Ridge were selected for this study (Fig. 1). Results indicate that Gakkel lavas can be sub-divided into primitive and evolved basalts based on their major and trace element compositions (Fig. 2). The distinction of the Gakkel basalts is based on MgO value at 8.5 wt%, as this is the inception point of decreasing MgO content along the liquid line of descent. Below this value a larger diversity of trace elements occurs in a global MORB compilation^57^, indicating an onset of sub-surface magma lens activity.

**Figure 3:**
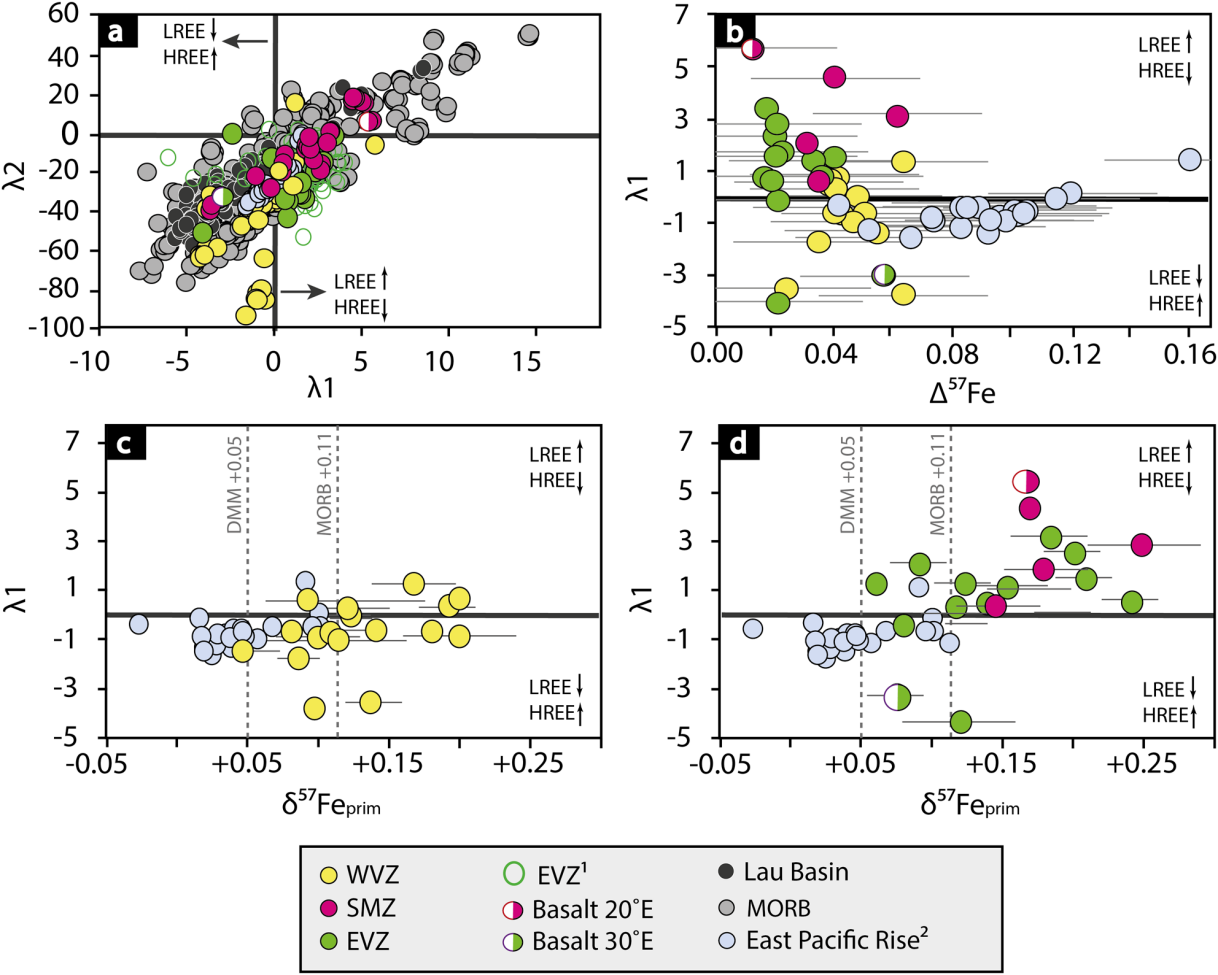
Rare Earth Element (REE) patterns versus δ^57^Fe_prim_. The REE patterns are given here as λ1 and λ2, describing the slope (λ1) and curvature (λ2) of the REE pattern. These parameter provide further information about the mantle source and petrogenesis^58^, which we link with iron isotope systematics. Lambda variables were calculated using the method of O’Neill^58^. λ1 (slope) can be translated to: λ1 > 0 indicates an enrichment of LREE over HREE, and λ1 < 0 represents the opposite with an enrichment of HREE over LREE. λ2 (curvature) marks the relative difference in LREE over HREE which captures the influence of mineralogy as a function of respective partitioning during fractionation or partial melting. Each basalt sample can have a different curvature for a given λ1^58^. (a) λ2 versus λ1 shows a clear distinction between the basalts from the WVZ and the SMZ, whereas basalts from the EVZ plot in between. MORB data is plotted here for comparison and was calculated from Jenner and O'Neill^57^. (**b**) REE patterns show a negative co-variation with increasing magmatic differentiation Δ^57^Fe. Basalts that experienced a lower degree of fractionation (Δ^57^Fe < 0.04 ‰) display a higher LREE content, compared to basalts that experienced a higher degree of fractionation (Δ^57^Fe > 0.04 ‰). (**c**) and (**d**) show λ1versus δ^57^Feprim for the slow spreading WVZ and the ultraslow spreading SMZ and EVZ. Basalts from the westernmost ridge segment (**c**) display homogenous REE pattern, which co-align with the data from the super-fast East Pacific Rise data^49^. In contrast, the SMZ and the EVZ lavas (**d**) from the ultraslow spreading ridge display a higher enrichment in LREE (λ1 > 0) compared to the WVZ and East Pacific Rise lavas from faster spreading regimes^49^. The two grey vertical dashed lines represent the iron isotope value of the depleted MORB mantle (DMM)^59^ and MORB^25,49^. 1—literature data for EVZ basalts are compiled from Gale et al.^2^. 2—literature data from the East Pacific Rise basalts are taken from Chen et al.^49^.

Primitive Gakkel basalts are characterised by a high MgO > 8.5 wt% and Ni concentration (up to 300 ppm), but also show moderate TiO_2_ content (< 1.5 wt%) and a slight depletion in heavy rare earth elements (HREE) and enrichment in light rare earth elements (LREE) (Fig. 3). Basalts of primitive character are mainly found in the EVZ and partially in the SMZ. In contrast, the more evolved Gakkel lavas show a MgO < 8.5 wt%, high TiO_2_ values (> 1.5 wt%) and moderate to high Ni concentration (< 150 ppm). The REE in those basalts show an enrichment in HREE over LREE, contrasting the observations made for the more primitive basalts (Fig. 3a). Gakkel samples with such characteristics are mainly found in the WVZ and partially in the SMZ.

Iron isotope systematics in the Gakkel Ridge basalts show no clear distinction between primitive and evolved basalt. The iron isotope composition of all analysed Gakkel basalts range in δ^57^Fe from + 0.10 to + 0.31 ‰ (Mg# = 55–69; Table 1). Basalts from the SMZ display isotopically heavier δ^57^Fe values than basalts from the WVZ and the EVZ. The average value for the SMZ lavas is + 0.22 ‰, whereby for basalts from the WVZ and the EVZ the average δ^57^Fe values are + 0.17 ‰ and + 0.16 ‰, respectively.

**Table 1:** Iron isotope data of the Gakkel Ridge basalts (whole rock) and volcanic glasses (VG) relative to IRMM-524A reference material.

Sample name	Ridge segment	Mg#	δ^56^Fe [‰]	± 2SE^#^	δ^57^Fe [‰]	± 2SE^#^	δ^57^Fe_prim_ [‰]	n	Δ^57^Fe [‰]
HLY0102 D011–19	WVZ	62.2	+ 0.11	0.01	+ 0.13	0.03	+ 0.09	3	0.04
HLY0102 D8–12	WVZ	61.8	+ 0.10	0.01	+ 0.12	0.02	+ 0.08	5	0.04
PS 66 217–16 (B)	WVZ	59.7	+ 0.13	0.02	+ 0.17	0.02	+ 0.12	7	0.05
PS 86–019 (B)	WVZ	62.1	+ 0.14	0.05	+ 0.23	0.02	+ 0.19	3	0.04
PS 86–67 (VG)	WVZ	60.7	+ 0.10	0.01	+ 0.15	0.03	+ 0.11	4	0.04
PS 86–044 (VG)	WVZ	61.3	+ 0.15	0.02	+ 0.24	0.01	+ 0.20	7	0.04
PS 59 216 (B)	WVZ	61.3	+ 0.16	0.01	+ 0.24	0.04	+ 0.20	5	0.04
Replicate			+ 0.16	0.04	+ 0.21	0.04	−	5	−
PS 59 216 (VG)*	WVZ		+ 0.13	0.03	+ 0.21	0.04	−	5	−
PS 59 216–11 (B)	WVZ	61.2	+ 0.09	0.02	+ 0.14	0.02	+ 0.10	7	0.04
PS 59 216–11 (VG)*	WVZ		+ 0.10	0.01	+ 0.14	0.02	−	4	−
PS 59 216–13 (B)	WVZ	61.4	+ 0.13	0.01	+ 0.18	0.01	+ 0.14	3	0.04
HLY0102 D013–10	WVZ	57.6	+ 0.08	0.01	+ 0.10	0.03	+ 0.05	3	0.06
PS 59 231–14	WVZ	54.8	+ 0.12	0.04	+ 0.16	0.01	+ 0.10	5	0.06
PS 59 232–84	WVZ	61.4	+ 0.09	0.02	+ 0.16	0.03	+ 0.12	3	0.04
HLY0102 D024–2	WVZ	63.3	+ 0.08	0.00	+ 0.12	0.03	+ 0.09	5	0.04
Replicate			+ 0.08	0.02	+ 0.12	0.03	−	6	−
HLY0102 D026–9	WVZ	59.7	+ 0.08	0.02	+ 0.16	0.04	+ 0.11	6	0.05
Replicate			+ 0.06	0.02	+ 0.10	0.03	−	6	−
HLY0102 D018–1	WVZ	58.6	+ 0.17	0.03	+ 0.23	0.01	+ 0.18	4	0.05
PS 59 234–24	WVZ	66.0	+ 0.11	0.02	+ 0.14	0.03	+ 0.14	3	0.02
PS 59 224–34	WVZ	55.6	+ 0.15	0.03	+ 0.23	0.02	+ 0.17	6	0.06
PS 59 243–36	SMZ	63.0	+ 0.13	0.01	+ 0.18	0.02	+ 0.14	7	0.04
HLY0102 D037–5	SMZ	55.8	+ 0.19	0.02	+ 0.31	0.04	+ 0.25	5	0.06
HLY0102 D036–35	SMZ	61.5	+ 0.15	0.01	+ 0.21	0.01	+ 0.17	4	0.04
HLY0102 D038–6	SMZ	64.0	+ 0.14	0.05	+ 0.21	0.03	+ 0.18	3	0.03
PS 59 251–4	SMZ	68.9	+ 0.12	0.01	+ 0.18	0.01	+ 0.17	4	0.01
PS 59 263–26	EVZ	57.4	+ 0.12	0.02	+ 0.13	0.02	+ 0.07	5	0.06
HLY0102 D050–30	EVZ	67.6	+ 0.14	0.05	+ 0.20	0.02	+ 0.18	3	0.02
HLY0102 D051–15	EVZ	66.8	+ 0.08	0.01	+ 0.11	0.02	+ 0.09	4	0.02
PS 101 186 R1 Basalt	EVZ	67.7	+ 0.12	0.02	+ 0.15	0.01	+ 0.14	4	0.02
PS 101 186 VG*	EVZ	67.4	+ 0.10	0.01	+ 0.13	0.01	+ 0.12	8	0.02
PS 59 294–40	EVZ	66.4	+ 0.09	0.02	+ 0.14	0.04	+ 0.12	4	0.02
PS 101 193 R1	EVZ	66.7	+ 0.10	0.01	+ 0.14	0.02	+ 0.12	4	0.02
PS 101 203 R4	EVZ	62.5	+ 0.07	0.01	+ 0.10	0.02	+ 0.06	4	0.04
PS 101 203 R5	EVZ	64.2	+ 0.14	0.02	+ 0.19	0.02	+ 0.15	5	0.03
PS 101 203 R8	EVZ	62.7	+ 0.17	0.03	+ 0.28	0.02	+ 0.24	5	0.04
PS 59 274–52	EVZ	66.4	+ 0.07	0.02	+ 0.10	0.01	+ 0.08	6	0.02
HLY0102 D059–35	EVZ	66.7	+ 0.14	0.01	+ 0.22	0.02	+ 0.20	7	0.02
HLY0102 D061–11	EVZ	66.6	+ 0.14	0.01	+ 0.23	0.02	+ 0.21	7	0.02

n = number of analyses; *VG-volcanic glasses, which are treated as sample replicates, because the VG and basalts are from the same rock sample. ^#^ Uncertainty is given as two standard error calculated from the number of analyses and the Student’s *t* correcting factor.

In order to assess the spread of Fe isotopes from the Gakkel Ridge MORBs, our results were compared to literature MORB values from slow-, intermediate- and fast spreading ridges, of which not many exist to date^25,28,49^; and to basalts from the Lau Basin back-arc basin^39,40^. The comparison with data from faster spreading ridges and back-arc basin mantle allows us to investigate (i) the role of spreading rate, tentatively related to sub-oceanic magma chambers and (ii) mantle source heterogeneity, such as metasomatic overprint or melt depletion as commonly found in subduction zones.

As melt fractionates *en route* to the surface, the ratio of ferrous (Fe^2+^) versus ferric (Fe^3+^) iron changes due to physical removal of a preferential Fe^2+^-species olivine^38^, which is accompanied by increasing δ^57^Fe^25,28,43,44,60^. The effect on Fe isotope systematics of crystal fractionation during melt ascent and source variability, in the Gakkel basalts, is assessed by modelling a putative, primary source. This is done by correcting for crystal fractionation. In this model, olivine is incrementally added to the melt until a composition of Mg#_melt_ = 74 (Fo_90_ in residue) is reached that is in chemical equilibrium with the mantle. This approach not only allows an approximation for the Fe isotope nature of the melts at mantle depth (expressed as δ^57^Fe_prim_), but also creates a calculated measure for isotope fractionation through crystal fractionation, expressed here as Δ^57^Fe = δ^57^Fe − δ^57^Fe_prim_^40,61^. If the difference between the primary source δ^57^Fe_prim_ and δ^57^Fe is large, the basaltic melt was strongly affected by crystal fractonation processes, whereas if the difference between both values is low, then the melt was fractionated to a minor extend. The applied correction method for the primary iron isotope composition has an estimated uncertainty of ± 0.05 ‰, which stems from the fractionation factor of iron between olivine and melt that is dependent on temperature and pressure^40,61^. Further uncertainty is added due to natural chemical variations in the upper mantle. A change of Mg# ± 2 would affect the iron isotope variation by ± 0.01 ‰^40^.

**Figure 4:**
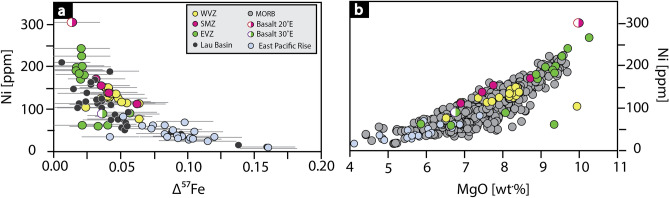
Crystal fractionation. Δ^57^Fe **(a)** and MgO **(b)** decreases exponentially with decreasing Ni content. This indicates that the modelling approach is valid, as both parameter (MgO and Δ^57^Fe) co-variate with Ni. Iron isotope data from the Lau Basin data are from Nebel et al.^40^ and East Pacific Rise from Chen et al.^49^. MORB data shown in (**b**) are from Jenner and O'Neill^57^.

As stated above, for the modelling, olivine is incrementally added to obtain the iron isotope composition of the ‘source’, which was originally removed by crystal fractionation. Olivine is a Mg-Fe-rich mineral that incorporates vast amounts of Ni in their crystal structure. By removing olivine from the parental melt Mg, Fe and Ni decreases with increasing crystal fractionation. This effect is shown in Fig. 4, validating the applied correction method. Nickel is plotted versus Δ^57^Fe (= expression for isotope fractionation) (Fig. 4a) and MgO (= control for crystal fractionation) (Fig. 4b), showing co-variations with both. With increasing fractionation (high Δ^57^Fe) and the removal of olivine, the Ni concentration must decrease, noting that Ni is an independent parameter. The figure reveals that the EVZ basalts have a low Δ^57^Fe (< 0.04 ‰), consistent with very limited igneous differentiation. Basalts from the WVZ display higher calculated Δ^57^Fe (> 0.04 ‰) values (Fig. 4a). Basalts from the SMZ show intermediate values between the EVZ and the WVZ. Literature samples from the East Pacific Rise^49^ follow the same trend, but exhibit a much larger effect of crystal fractionation.

**Figure 5:**
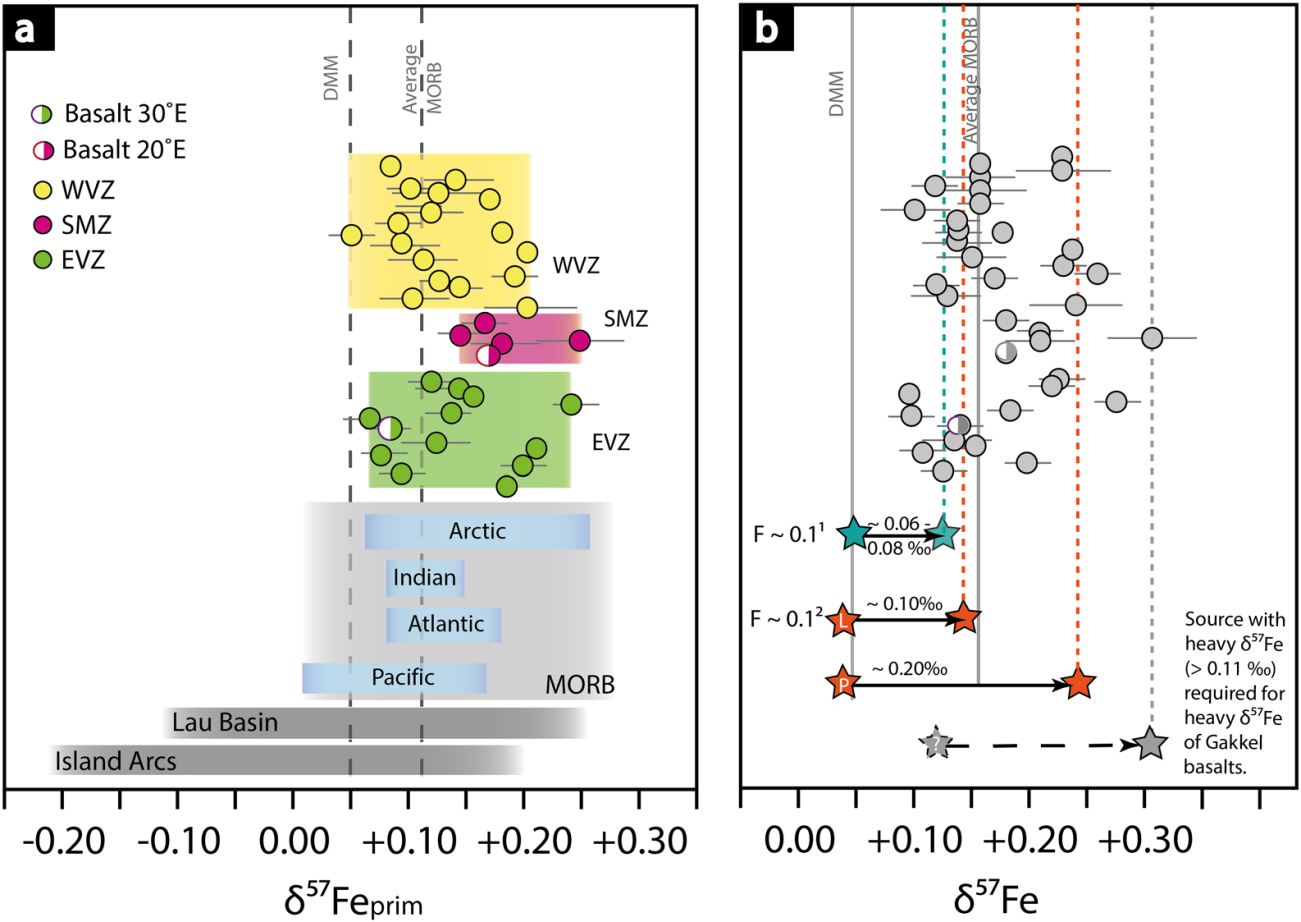
Primitive Fe isotope composition and overview of partial melting models. **(a)** Comparison of δ^57^Fe_prim_ from different locations and tectonic settings. The different colours highlight different ridge segments, locations and tectonic settings. The δ^57^Fe_prim_ composition of MORBs was calculated from Teng et al.^25^. The depleted MORB mantle-value is given as reference^59^. Lau Basin and island arc data are compiled from Nebel et al.^40^ and Foden et al.^42^. Arctic ridge data shown in the grey box, represents the overall variability in Gakkel basalts from this study. **(b)** δ^57^Fe of the Gakkel basalts with an overlay of melt and initial δ^57^Fe compositions derived from partial melting models^42,62^. The stars connected by an arrow show the initial δ^57^Fe composition of the source and the corresponding melt at 10% partial melting. 1—In Foden et al.^42^ the depleted MORB mantle with an initial δ^57^Fe of + 0.05 modelled, which produce melts between 0.11 and 0.13. 2—Williams and Bizimis^62^ showed that lherzolite (L) and pyroxenite (P) with an initial δ^57^Fe of 0.04 ‰ can produce corresponding melts with δ^57^Fe increasing in 0.1–0.25 ‰, respectively. To allow for the heavy δ^57^Fe of the Gakkel Ridge (> 0.20 ‰) the source needs to be isotopically heavier than the depleted MORB mantle. The grey star at δ^57^Fe = 0.11 ‰ marks a hypothetical source composition for the heaviest Gakkel basalts. The heaviest δ^57^Fe composition of abyssal peridotites, which are considered as the melting residue of MORBs, from the Gakkel Ridge have a value of ~ 0.10 ‰ (LOI < 1.0 wt.%)^59^. To achieve a source with such values, it is likely that the source requires a higher modal composition in pyroxene or is of metasomatic origin.

Figure 5 shows the ‘*primitive*’ Fe isotope composition of the Gakkel MORBs (Mg# = 55–70) ranging over 0.20 ‰ in δ^57^Fe_prim_. These variations are slightly higher compared to global MORBs (range in 0.15 ‰ in δ^57^Fe_prim_) and smaller compared to basalts from the Lau Basin, which show a large range of 0.35 ‰ δ^57^Fe_prim_^40^, and global arc basalts with extreme variations over 0.40 ‰ δ^57^Fe_prim_, ranging from -0.21 to + 0.20 ‰^42^.

## Discussion

### The effect of fractional crystallisation on Fe isotope composition

The Gakkel Ridge in the Arctic Ocean is classified as an ultraslow spreading ridge based on its distinct characteristics such as spreading rate < 12 mm/a, the existence of amagmatic ridge segment, no transform fault and low magma supply due to lower mantle potential temperatures^1,50,63^. The westernmost segment at the Gakkel Ridge, the WVZ, however, displays characteristics of slow spreading ridges (> 12 mm/a). It is likely that the WVZ reflects a transition between ultraslow and slow spreading ridges, which is a function of magma supply, ridge geometry and mantle temperature^1,64^. The higher magmatic activity^50,63^ and thicker oceanic crust^63^ observed at the WVZ could be related to higher mantle temperatures beneath the ridge segment, which results in an increased melt production and promotes the formation of a magma chambers or melt lenses^10^. In contrast, the SMZ and the EVZ are described to have a low magmatic activity, which could results from lower potential mantle temperatures. It is thus plausible that the magma plumbing system beneath the Gakkel Ridge is divided into two different systems dependent on their spreading rate.

Recent studies on the modal composition of phenocrysts in MORBs and variable textures in plagioclase in basalts from the Gakkel Ridge suggest that the ascending melt travels through crystal mush zones in which melt replenishment and mixing occurs^6,22,65^. Combining these petrographic observations with Fe isotope systematics from the Gakkel Ridge suggests that the observed iron isotope fractionation of Δ^57^Fe < 0.04 ‰ could be indeed linked to the occurrence of smaller crystal mush zones in areas with low magmatic activity (EVZ, SMZ), whereas in areas with higher magmatic area (WVZ) these mush zones have a greater extent enhancing iron isotope fractionation (Δ^57^Fe > 0.04 ‰). The ascribed processes presented here are conform to petrographic observation^6,22^. Even though the Fe isotope systematics associated with crystal fractionation (Δ^57^Fe) are small, they follow petrogenetic evidence and systematics, and are thus interpreted here to reflect crystal fractionation processes. Accordingly, the occurrence of magma lenses beneath the active ridge appears to translate into Fe isotope variations, due to enhanced fractional crystallisation resulting in a higher iron isotopic fractionation and thus a higher calculated Δ^57^Fe. In analogy, the same systematic would apply to other MORBs.

**Figure 6:**
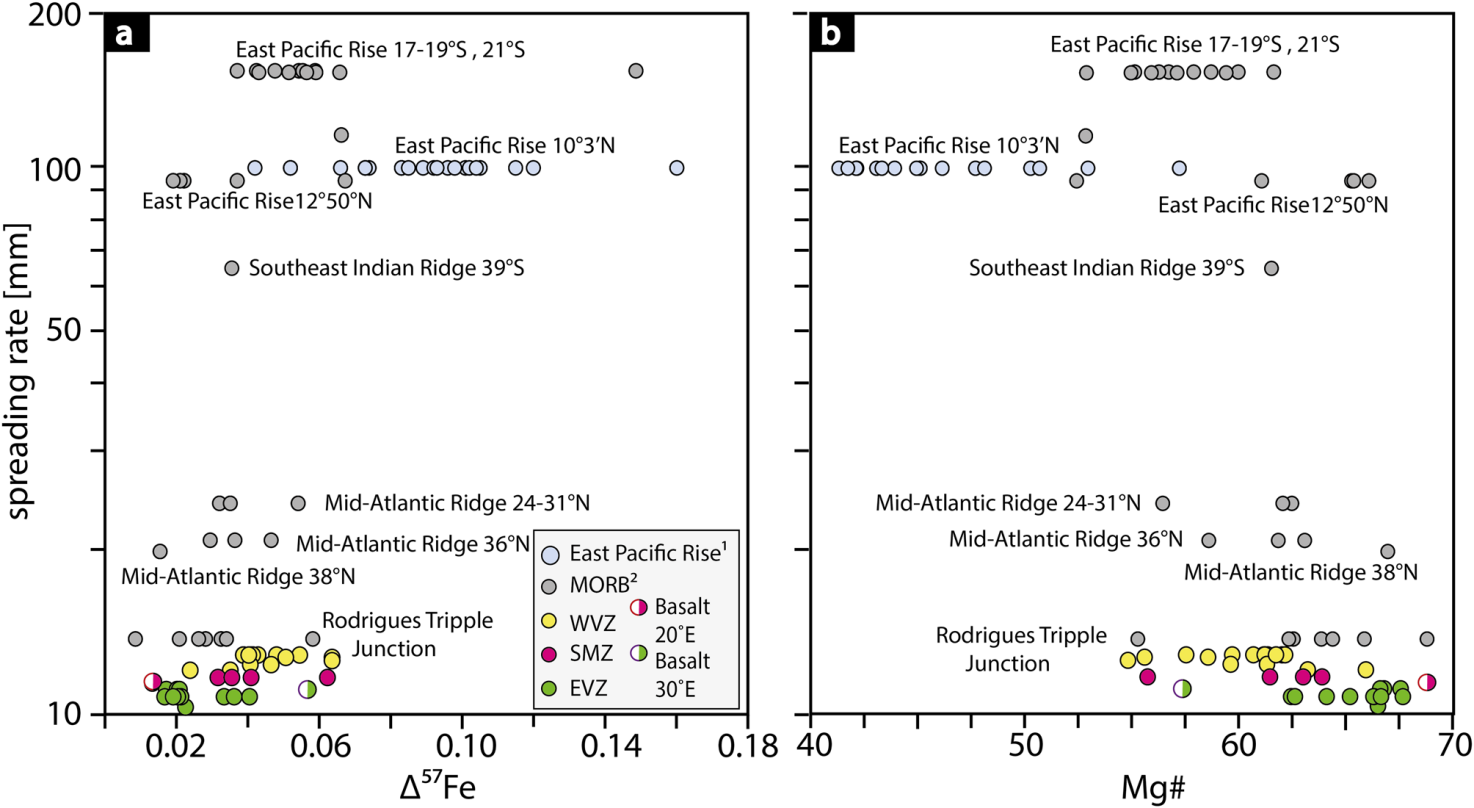
The effect of spreading rate on Δ^57^Fe and Mg#. **(a)** Δ^57^Fe co-varies positively with increasing spreading rate and Mg# **(b)** decreases with increasing spreading rate, in particular within the Gakkel Ridge basalts. 1—Iron isotope data from the East Pacific Rise are compiled from Chen et al.^49^. 2—Iron isotope data for MORBs are taken from Teng et al.^21^.

**Figure 7:**
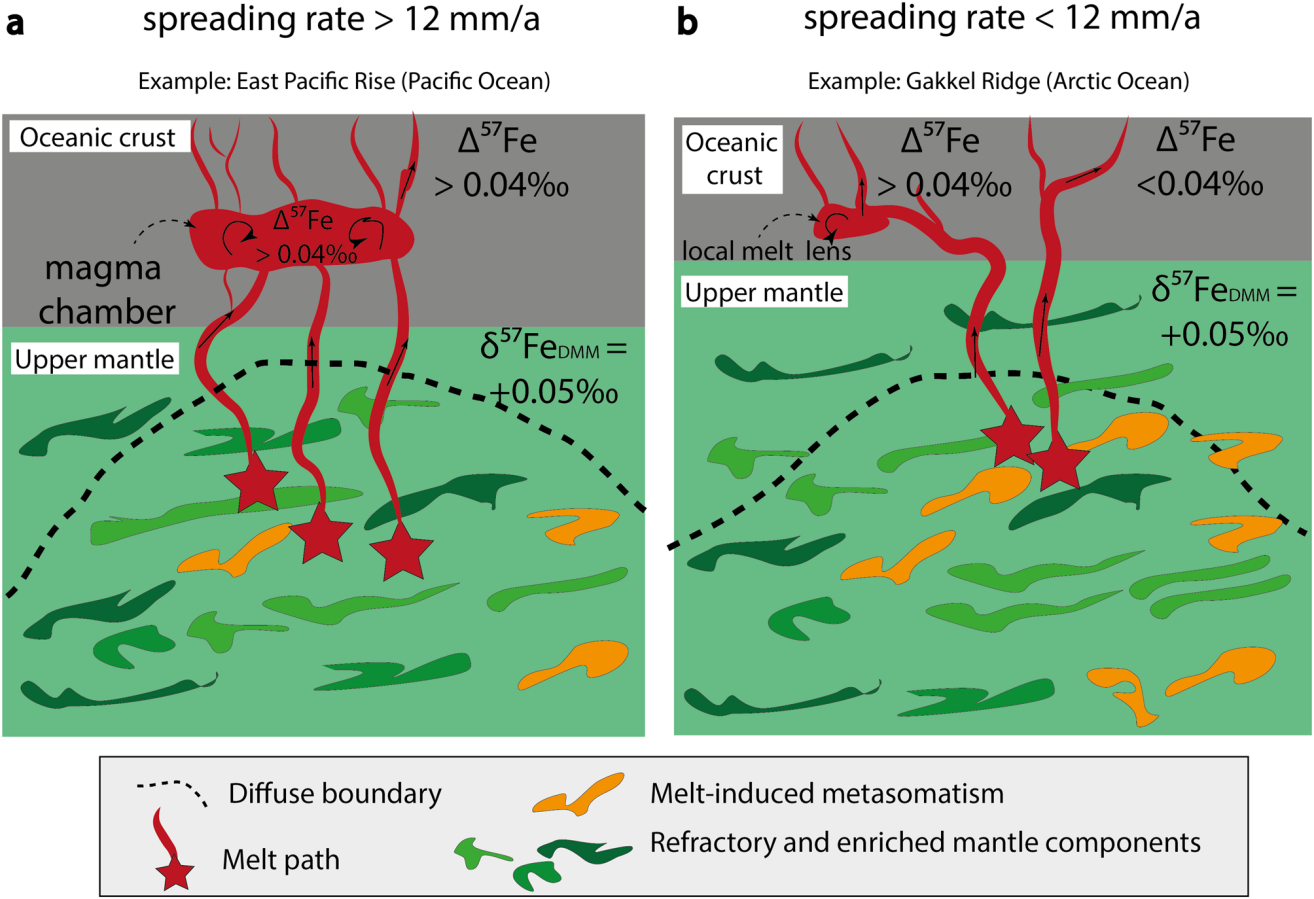
Magma plumbing systems at the Gakkel Ridge. Simplified sketch after Brandl et al.^3^ and Nebel et al.^39^, showing magmatic evolution depending on spreading rate differences. **Profile A** reflects the melt migration and the formation of large mid-crustal magma chambers at spreading rates > 12 mm/a. Melt migrating through magma chambers will be controlled by magma mixing and crystal fractionation affecting the iron isotope composition of the basalts (Δ^57^Fe > 0.04 ‰). Instead, **profile B** represents the magmatic process for ridge with a rate of < 12 mm/a. In this case, magma travels from its source region to its surface without or minor interaction in magma pools, which is reflected by lower degree of isotope fractionation (Δ^57^Fe < 0.04 ‰).

Basalts from the WVZ indicate a slightly stronger control of crystal fractionation (Δ^57^Fe > 0.04 ‰; lower Mg# 55–62) compared to basalts from the EVZ and the SMZ (Δ^57^Fe < 0.04 ‰; higher Mg# 57–67). This slight difference is consistent with the small variation in spreading rate (Fig. 6). We suggest that melts at the WVZ travel through a mid-crustal magma lens or magma reservoir (Fig. 7 profile A) in which the melt experienced crystal fractionation. In contrast, samples from the SMZ and the EVZ are from an amagmatic area of low magmatic activity with a thin oceanic crust^1,50,63,66^. All listed characteristics in the SMZ and the EVZ are typical for ultraslow spreading rates^1,3,10,50^. The low magmatic activity, in particular in the EVZ, may stem from potentially colder upper mantle conditions^67,68^. Thus, cooler mantle condition may cause the formation of a permeability barrier in deeper levels (i.e., in the upper mantle), where melts may pool^69,70^. From these melt pools, melt can ascend rapidly to the crust with minor crystal fractionation or wall-rock-interaction, and preserve the primitive nature of the melt (Fig. 7 profile B). The rapid melt transport from upper mantle levels is supported by barely resolvable Fe isotope fractionation corrections (Δ^57^Fe < 0.04 ‰), a fact not surprising given their high MgO content (> 8.5 wt%). Notable is that basalts from the EVZ show high Mg# and no co-variation of trace elements (V/Sc, REE) with increasing isotopic fractionation (Figs. 2d, 3b,d). We therefore suggest that the Fe isotope composition of the EVZ basalts closely resemble those of their mantle source with only minor crystal fractionation, wall-rock-interaction, and most likely no homogenization in sub-oceanic magma lenses. All other samples appear to be, at least in parts, affected by crystal fractionation.

Such process is expected to be more pronounced for faster spreading ridges, which can, indeed, be observed if the Gakkel samples are compared to *super-*fast spreading East Pacific Rise ridge samples, showing higher calculated Δ^57^Fe (Figs. 3, 6). The high Δ^57^Fe in the East Pacific Rise lavas is in line with a strong influence of crystal fractionation in these samples^49^. Despite experimental evidence that equilibrium olivine crystal fractionation is not responsible for Fe isotope variations in evolving melts^71^, proxies of crystal fractionation in these natural systems can only plausibly be explained by the removal of isotopically light Fe, linked here to olivine on the basis of co-variations with Ni and MgO.

Figure 7 shows the two different plumbing systems that are present at the Gakkel ridge and possibly apply for ridges elsewhere: (1) direct melt transport through dikes from the source region to the crust at spreading rates < 12 mm/a, represented here by Δ^57^Fe < 0.04 ‰ and > 8.5 wt% MgO, and present at the SMZ and the EVZ; (Fig. 7b) and (2) melt transport through magma lenses at spreading rates > 12 mm/a, characterized by Δ^57^Fe > 0.04 ‰ and < 8.5 wt% MgO, and represented by basalts from the WVZ (Fig. 7a).

#### Outlier from magmatic trends

Two basalts, one at 20°E and one at 30°E outlie from their respective ridge segment. The first sample at 20°E is from the SMZ (PS 59 251–4; Δ^57^Fe = 0.01 ‰) shows an extremely high Mg#, Ni, TiO_2_ and V/Sc ratio (Figs. 2c,d; 4), which is in contrast to other mid-ocean ridge lavas from the same ridge segment. The high Mg# and Ni content reflects a primary melt composition with a source that may contain higher modal olivine. The higher TiO_2_ concentration and V/Sc in this sample, however, may suggest a higher modal amount of pyroxene, because Ti and V are preferably incorporated in pyroxene. It is possible that this sample may be affected by pyroxenite in its mantle source, whereby an excess of Si enhanced preferential orthopyroxene formation that is associated with the release of Ni^72^. Alternatively, this sample represents an oceanic cumulate layer, which typically occurs in magma chambers or in small local magma lenses at lower crustal level^73^. Melt ascending from lower crustal levels to the surface does not undergo much crystal fractionation or wall rock interaction, due to a rapid melt transport. The latter would explain the virtually absence of isotope fractionation (Δ^57^Fe of 0.01 ‰ calculated for this sample).

The second sample that outlie from their respective sample set is the basalt at 30°E (PS 59 263–26) from the EVZ, which shows a higher degree of fractionation (Δ^57^Fe = 0.06 ‰) compared to other basalts from the same ridge segment which show values below 0.04 ‰ (Figs. 2, 4). This sample shares the chemical properties (low Mg# and high Δ^57^Fe) with basalts from the WVZ. We, therefore, suggest that the melt migrates through an isolated melt lens at crustal levels, where crystal fractionation is a dominant process effecting the melt chemistry.

### Mantle source variability at the Gakkel Ridge

As discussed in the section above, crystal fractionation affects the Fe isotope composition of the Gakkel basalts by no more than 0.06 ‰, which is a considerable small crystal fractionation effect on δ^57^Fe. The measured Fe isotope composition (uncorrected values) of the Gakkel lavas, however, range by over 0.20 ‰ δ^57^Fe, from + 0.09 to + 0.31 ‰ (Fig. 5), which is surprisingly similar to the existing Fe isotope data from the fast spreading East Pacific Rise ridge that show a much greater effect of crystal fractionation on δ^57^Fe (Figs. 2 and 3). Consideration of minor Fe isotope fractionation observed in the Gakkel lavas during crystal fractionation and the wide δ^57^Fe range of 0.20 ‰ must require an inherited variability from the source region. Such isotopic variability can either be induced through partial melting processes or inheritance from an isotopically diverse mantle.

In the following, we assess the extent to which mantle heterogeneity affect the iron isotope composition by combining δ^57^Fe_prim_ with REE pattern of the Arctic mid-ocean ridge basalts (Fig. 3). The REE of each sample is expressed, here, in lambda notations (λ0, λ1, λ2, λ3, λ4), which reflects the chondrite-normalized REE pattern as a polynomial^58^. Each lambda notation expresses the REE pattern shapes (average, slope, curvature, shape) of all 14 measured rare earth elements as a single value. This is a powerful tool, because the lambda notation is able to reflect a variety of REE pattern shapes, which are linked to their respective parental source^58^. Lambda 1 reflects the slope and λ2 the curvature of the REE pattern; both combined are particularly useful to investigate the nature of the mantle at the Gakkel Ridge in combination with the corrected Fe isotope values (δ^57^Fe_prim_), which provides an estimate of the parental source composition. This allows for a better comparison with the East Pacific Rise data to assess the effects of mantle source heterogeneity and spreading rate on Fe isotope data. At this point, we would like to highlight that for the Gakkel data alone, the non-corrected δ^57^Fe values lead to similar conclusions.

Apart from crystal fractionation and mantle source, partial mantle melting is an additional process that contributes to iron isotope compositions in lava. Previous studies show that partial mantle melting leads to heavier Fe isotope signatures in melts^42,61^. An average MORB has a partial melting degree of 15–20% and this would only account for an 0.06 ‰ increase in δ^57^Fe values for either, equilibrium or batch melting^42,61^. The enrichment in heavy Fe isotopes by only 0.06 ‰ cannot explain the high δ^57^Fe values observed at the Gakkel Ridge (Fig. 5b). Likewise, it cannot account for the chondritic fractionation-corrected Fe isotope values of the East Pacific Rise, which are too light compared to MORB elsewhere^25,61^. In the following, we focus on the effects of mantle source heterogeneity and spreading rate on Fe isotope data.

#### Ultraslow eastern volcanic zone vs ultrafast east pacific rise

The Eastern Volcanic Zone (EVZ) displays the slowest spreading rates (< 10 mm/a) of the entire the Gakkel Ridge and is characterised by a large range in δ^57^Fe_prim_ =  + 0.06 to + 0.24 ‰ (Fig. 5a). The variation in δ^57^Fe_prim_ does not only occur along the entire ridge segment, but also in samples from a single dredge. At 61°E, the Fe isotope composition ranges from *depleted MORB mantle*-like values to highly enriched mantle values (Fig. 3), implying that chemical heterogeneities must exist on a smaller scale (km–range). Magma mixing and mingling in small crystal mush zones potentially erases such heterogeneity^4^, as for basalts from faster spreading segments (> 12 mm/a).

When corrected to a single mantle source, and with a homogeneous mantle source assumed, all data should cluster around δ^57^Fe_prim_ =  ~  + 0.10 ‰, as shown by Sossi et al.^61^, who corrected global MORB data by Teng et al.^25^ for crystal fractionation. The Gakkel data, however, ranges from chondritic values at δ^57^Fe_prim_ =  + 0.05 to + 0.24 ‰, with the majority between δ^57^Fe_prim_ =  + 0.10 to + 0.15 ‰ (Fig. 5a). In contrast, fractionation corrected data for the East Pacific Rise shows that the primitive Fe isotope compositions of nearly all samples are near-chondritic (Fig. 3c). Allowing for Fe isotope fractionation during partial melting^42,61^ demands that the East Pacific Rise source is either sub-chondritic in composition, or that the effect of partial melting on Fe isotopes is minimised or effectively absent. The latter seems most plausible, as to the fast spreading effect, allowing for a larger degree of melting, and thereby smaller isotope effects during partial melting. The Gakkel data is on average heavier than their Pacific counterparts, and this may be linked to the slow spreading rate (Fig. 5). Following the modelling outlined in Sossi et al.^61^ and Foden et al.^42^, partial melting of the mantle with *F* at ultraslow spreading ridges can be as low as 10% (*F* = 0.1), resulting in a fractionation of circa 0.06–0.08 ‰, and thereby accounting for some of the heavier data in Gakkel lavas (Fig. 5b). In contrast, fast spreading centres with *F* > 0.25 would experience no resolvable isotope fractionation^61^. Support comes from the fact that the WVZ samples trend towards East Pacific Rise corrected data, whilst the EVZ samples are isotopically heavier, aligned with their difference in spreading rate. A direct comparison of spreading rate, and associated degrees of melting therefore suggests that the geodynamic setting of the ridge directly influences the Fe isotope compaction of erupting lavas. At ultraslow spreading ridges, rapid ascend without magma chamber homogenisation further promotes the preservation of source compositions, whereas at fast spreading ridges, the larger magma supply and high *F* results in more pronounced crystal fractionation.

Crystal fractionation at fast spreading centres (Figs. 2, 3, 4) and low degree melting (Fig. 5b) are thus the controlling factors in driving Fe isotopes towards heavier values in erupting melts. Crystal fractionation affect the iron isotope composition to up to 0.15 ‰ at the ultrafast spreading East Pacific Rise, as shown in Figs. 2–4, whereas low degree melts (F = 0.1), which is predominant at ultraslow spreading rates, can affect the iron isotope composition of up to 0.08‰ (depleted MORB mantle)^42^ or 0.20 ‰ (pyroxenite)^62^, depending on the source composition. Erupted lavas from fast versus ultraslow spreading ridges would therefore appear similar in their absolute Fe isotope signatures, despite being controlled by different processes.

#### Excess heavy Fe isotopes at the Gakkel Ridge

As outlined above, basalts from the WVZ and the SMZ differ in their major and trace element compositions (Fig. 3). The WVZ basalts ranges from depleted MORB mantle values of δ^57^Fe_prim_ =  + 0.05 ‰ to heavy values of + 0.20 ‰, whereby the SMZ basalt range from + 0.15 to + 0.26 ‰. It appears that both ridge segments share components of a similar enriched source due to an overlap in the heavier isotope range and gradual change in the rare earth element pattern (Fig. 3c). At least four of the EVZ samples are also isotopically heavy with δ^57^Fe_prim_ >  + 0.15 ‰ (Fig. 5), which cannot plausibly be explained by partial melting of a depleted MORB mantle source (Fig. 5b).

Basalts from the SMZ and the EVZ are enriched in LREE (λ1 > 0), whereby the WVZ basalts tend to be more depleted in LREE (λ1 < 0). The decrease in LREE concentration from the SMZ and the EVZ to the WVZ is opposite of what is expected to occur during partial melting in the upper mantle (Fig. 3c,d), with crystal fractionation processes in magma lenses or crystal mush zones (Fig. 3b) excluded in the previous section. The only plausible explanation of these heavy Fe isotopes is mantle source enrichment.

Co-variations of REE pattern with δ^57^Fe_prim_ (Figs. 3c and 5b), the latter being interpreted here as the isotope signature of the mantle source, suggest that values of δ^57^Fe_prim_ >  + 0.15 ‰ is inherited from a geochemical diverse mantle source. The isotopically heavy δ^57^Fe_prim_ (> + 0.15 ‰) in the Gakkel Ridge basalts exceed values for the reported average MORB values and are likely due to an enrichment of heavy Fe isotopes in the mantle. The enrichment in heavy Fe isotopes requires a physical component added to the mantle source of the basalts^39,40,42,59^, or a source with a higher modal composition of pyroxene^62^. Experimental and field studies have shown that small modal amounts (< 4%) of pyroxenite within a MORB source can significantly affect the trace element budget of extracted melts, in particular LREE^20,74–76^. Enriched MORB sources with heavy Fe isotopes were reported from the Galapagos spreading centre, albeit in conjunction with plume-ridge interaction^77^. Melting of enriched mantle domains associated with a crustal component in the mantle can result in equally high δ^57^Fe (Fig. 7). Metasomatic enrichment of the mantle would have occurred prior to mid-ocean ridge genesis, since heavy iron isotope signatures are not prevalent in all Gakkel Ridge segments, in line with a heterogeneous geochemical character of the mantle below the Gakkel Ridge reported in previous studies^52,55^.

Intriguing is that previous studies^54,55^ reported mantle sections that are extremely depleted at approximately 65°E. Melt-depleted mantle at convergent margins show δ^57^Fe <  + 0.05 ‰, but the EVZ basalts, however, do not show such light values in δ^57^Fe_prim_ (Fig. 2). It remains unclear why the extremely depleted signatures observed at the EVZ are not reflected in Fe isotopes. Such depletion may thus be either of localised nature, and a rather unusual, rare find, or, more likely, does not contribute substantially to melt extruded at the ridge above^78^. As such, if ultra-depleted mantle lithologies are more abundant they bear no effect on δ^57^Fe, noting that sample bias is also possible.

## Concluding remarks

This study assesses the Fe isotope systematics at the Gakkel Ridge, focussing on differences in magmatic differentiation along-ridge with increasing spreading rate. Iron isotope modelling indicates that crystal fractionation (monitored through Δ^57^Fe) effects are resolvable but small with δ^57^Fe variations of only up to ~  + 0.06 ‰ (model dependent) at slow spreading rates (< 12 mm/a). This process is ascribed here to the development of magma reservoirs or crystal mush zones in mid-crustal levels, in which melt undergoes magma mixing and fractionation of olivine and clinopyroxene. The exact nature of this process needs to be further explored (e.g., through fractionation and diffusion experiments). However, it seems apparent that olivine is responsible for this effect, evidenced through co-variations with Ni abundances, and in line with observations of the fast spreading East Pacific Rise, where these effects are excelled^49^.

Correction for crystal fractionation indicate that basalts from ultraslow ridge segments show, on average, heavier values in the source than fast spreading ridges, which can plausibly be ascribed to effects of partial melting. With estimated F ~ 0.1 at ultraslow spreading ridges, ca. 0.06–0.08 ‰ heavier values can be observed^42,61^. The *super-*fast East Pacific Rise ridge^49^, however, shows chondritic values with virtually no Fe isotope fractionation during partial melting, implying F > 0.25^42,61^.

These processes, however, cannot explain the variability in Fe isotopes at the Gakkel Ridge, which is argued here to result from mantle source variability. This implies that the Gakkel Ridge lavas extend the existing δ^57^Fe MORB database towards heavier values and demonstrate that MORB sources can contain mantle components enriched in heavy Fe isotopes, e.g., pyroxenite,^79^. Indeed, these potentially contribute to geochemical variation (especially REE) in mid-ocean ridge basalts, as suggested in previous studies^20,74,75,78^. The reason for the absence of these signatures elsewhere maybe their admixture to a larger melt ponds in sub-oceanic melt lenses at faster spreading ridges. Alternatively, they are unique to the Gakkel Ridge with a known complex mantle source history^51,54,55^.

## Methods

### Samples

In total 35 dredged basalts from the three tectonomagmatic ridge segments were analysed to investigate the Fe isotope variability in MORBs. Twenty two basalts from the WVZ and the SMZ with known major and trace elements systematics were chosen for this study^53^. Additionally, thirteen basalts from the EVZ were crushed in a ceramic jaw crusher, milled in an agate mill to fine powder and analysed for their major and trace element compositions (Supplementary Data Table S1). The sample locality of all 35 samples, as well as the major and trace element data are provided in Supplementary Data Table S2. The samples analysed in this study are fresh and show no visible alteration. Loss on ignition (LOI) was determined and displays a range between -0.6 and + 3.11 wt%, with the majority of the samples being below + 1.0 wt%. Low LOI values, ranging between − 1.0 and + 1.5 wt.% indicate minor if any alteration.

### Major and trace elements

Major element compositions of the basalts were determined by admixing 0.5 g of sample whole-rock powder with 12:22 Li metaborate and lithium tetraborate flux. The mixture was fused using a Claisse M4 fluxer. The fused sample disc was analysed using a PANanalytical Axios Advanced WDS X-ray fluorescence (XRF) instrument coupled with a 4000 W Rh X-ray tube at the School of Earth Sciences, University of Tasmania. Fragments of each fused sample disc were mounted in 1-inch epoxy plugs and measured for the trace element content using laser ablation inductively coupled plasma mass spectrometer (LA-ICP-MS) at the Isotopia Laboratory at the School of Earth, Atmosphere and Environment, Monash University. Each analysed disc fragment was a triangular shape and represented the entire fused sample disc from the centre to the rim. Four to five spots were measured on each fused disc fragment with spots distributing from the centre to the rim to exclude a potential heterogeneity within the fused disc. During the LA-ICP-MS analysis forty-three elements (Na, Mg, Al, Si, Ca, Sc, Ti, V, Cr, Mn, Fe, Co, Ni, Cu, Zn, Ga, Rb, Sr, Y, Zr, Nb, Mo, Cs, Ba, La, Ce, Pr, Nd, Sm, Eu, Gd, Tb, Dy, Ho, Er, Tm, Yb, Lu, Hf, Pb, Th, U) were determined using an ASI RESOlution SE (S-155) 193 nm ArF excimer laser coupled to a ThermoFisher Scientific ICapQ. Spot size, repetition rate and energy density were set to 80 µm, 10 Hz and 3.0 J/cm^2^, respectively. One single analysis comprises of 20 s background signal, 30 s data acquisition and 20 s washout. The integration time for the isotopes from Na–Ga was set to 1 ms, for Rb- Sm as well as for Hf, Pb, Th and U to 2 ms, and for elements from Eu–Lu to 3 ms, respectively. Data were acquired in a time-resolved analysis mode and reduced using a Microsoft Excel spreadsheet developed at the Max Planck Institute for Chemistry (MPIC) Mainz, Germany^80,81^. BCR2G (basaltic reference glass) was used as calibration standard and Si (determined by XRF) as internal standard. For data control the reference glasses NIST 610, NIST 612, BCR2G and BHVO2G were bracketed after blocks of twenty unknowns. The elemental concentration of each sample bases on the average composition of all measured spots on each fragment. An XRF fused glass blank was analysed with the fused sample glasses to account for potential contamination of the fusion process for major element analysis. The glass blank made out of high purity glass contains less than 1 ppm of trace element. The obtained trace element data of the basalts from this study is consistent with Gakkel Ridge data presented by Gale et al.^2^. The average precision for the matrix-matched reference material (BHVO2) of all forty-two measured elements is ~ 1%. Data for each element and reference material is provided in Supplementary Data Table S1.

### Iron isotopes

Iron isotope analysis (expressed as δ^57^Fe) was carried out in the Isotopia Laboratory at the School of Earth, Atmosphere and Environment, Monash University. Circa 100 mg of rock powder were dissolved in a mix of concentrated HF:HNO_3_ (ratio 1:5) and left for 24 h on a hotplate at 120 °C until no macroscopic residue was observed. After the digestion and evaporation, the sample solutions were dried down three times, after adding three drops of concentrated HNO_3_ and two drops of a weak HCl-HF mixture to break the silica-calcium bonding. Samples were taken up in 2 ml of 9 M HCl, left overnight on a hotplate at 100 °C and were centrifuged, before chemical separation of Fe from the rock matrix. For the Fe purification the anion resin AG1-X-8 resin (Bio-Rad 200–400 μm mesh) was used to remove the matrix following the chromatographic procedure of Sossi et al.^82^ and Cheng et al.^83^. The extracted Fe was introduced into the Ar plasma using a Thermo Scientific quartz cyclonic spray chamber. All samples were analysed in 2% HNO_3_ using a Neptune plus MC-ICP-MS with ^53^Cr, ^54^Fe, ^56^Fe, ^57^Fe, ^58^Fe, ^60^Ni and ^61^Ni ions measured simultaneously on Faraday cups (L4, L2, L1, C, H1, H2, H4) at medium resolution using the standard-sample bracketing method^84^. Iron isotope data is reported relative to the international Fe isotope standard IRMM-524A, which is isotopically identical to IRMM-014^85^, and given in the delta notation:

$$\delta^{{\text{X}}} {\text{Fe}}\left( {{\text{IRMM}} - {\text{524A}}} \right)\, = \,\left[ {\left( {^{{\text{X}}} {\text{Fe}}/^{{{54}}} {\text{Fe}}_{{{\text{sample}}}} } \right)/\left( {^{{\text{X}}} {\text{Fe}}/^{{{54}}} {\text{Fe}}_{{{\text{IRMM}} - {\text{524A}}}} } \right) \, - {1}} \right)] \times {1}000;$$

where ^X^Fe refers to ^57^Fe and ^56^Fe, respectively, and ^X^Fe/^54^Fe_IRMM-524A_ is calculated as average from the bracketing standard values. Instrumental mass bias was further corrected in each analytical session by using a Ni ICP standard solution using the exponential fractionation law and assuming equal mass biases of Fe and Ni^86^. All analyses were carried out with a Fe_IRMM-524A_ standard solution 5 ppm Fe and 15 ppm Ni resulting in a ~ 700 mV for ^57^Fe. The Ni solution was admixed to the IRMM-524A Fe standard solution (1:3 ratio) and sample prior to isotope analyses. Six procedural blanks were measured alongside the samples. Four blanks yielded 1 pg Fe and two blanks ranged between 25 and 47 pg. Each sample was measured between three and eight times.

Iron isotopes are reported as δ^57^Fe and δ^57^Fe_prim_ in this study. The Fe isotope values (δ^57^Fe) corresponds to the measured value of the rock, whereas δ^57^Fe_prim_ is the corrected value that represents the Fe isotope composition of the primitive melt in equilibrium with the mantle. A detailed description of the calculation for δ^57^Fe_prim_ is given in Sossi et al.^61^ and Nebel et al.^40^. Notable is that the correction applied here is employing a fractionation factor between olivine and melt, which can only act as a broad approximation, and are model parameter dependent. The uncertainty of the Fe isotope values are given as 2SE (standard error) in this contribution and the reproducibility of the analysis was tested by measuring replicates and rock reference material BHVO1 and BCR1. All measured replicates are within uncertainty of the sample. BHVO1 yielded on average a δ^56^Fe and δ^57^Fe of + 0.10 ± 0.02 ‰ and + 0.15 ± 0.03 ‰ (n = 12; 2SE), respectively, and BCR1 in a δ^56^Fe =  + 0.07 ± 0.02‰ and δ^57^Fe =  + 0.10 ± 0.03 ‰ (n = 12; 2SE). Both reference materials are within analytical uncertainty of published literature values^42,85,87^. The long term reproducibility of BHVO1 and BCR1 at the Monash Isotopia Laboratory is for δ^56^Fe =  + 0.10 ± 0.02‰ and δ^57^Fe =  + 0.16 ± 0.02 ‰ (n = 32, 2SE) and δ^56^Fe =  + 0.08 ± 0.01 ‰ and δ^57^Fe =  + 0.13 ± 0.02 ‰ (n = 35, 2SE), respectively. We, however, ascribe a ± 0.03 ‰ external reproducibility to all analyses in case of a smaller in-run precision. Data for the reference material is given Supplementary Data Table S3.

## Supplementary Information

Supplementary Information 1.

Supplementary Information 2.
