# Supplementary material for: Competing effects of spreading rate, crystal fractionation and source variability on Fe isotope systematics in mid-ocean ridge lavas
Source: Sci Rep. 2021 Feb 18;11:4123. doi: 10.1038/s41598-021-83387-7 (PMC7893168; doi:10.1038/s41598-021-83387-7)
Supplement: Supplementary file 2 — Supplementary Information 2. [file 41598_2021_83387_MOESM2_ESM.pdf]

Competing effects of spreading rate, crystal fractionation and source variability on Fe isotope systematics in mid-ocean ridge lavas

Marianne Richter[1], Oliver Nebel[1], Martin Schwindinger[1], Yona Nebel-Jacobsen[1] and Henry J.B. Dick[2]

[1] – Isotopia Laboratory, School of Earth, Atmosphere and Environment, Monash University, Clayton, Victoria, 3800, Australia

[2] – Department of Geology and Geophysics, Woods Hole Oceanographic Institution, Woods Hole, Massachusetts, 02543-1539, USA

Supplementary Data Table S2 - Sample location, major and trace elements of the Gakkel ridge basalts.

Sample Name	Ridge segment	Latitude	Longitude	Spreading rate[1] [mm/a]	CaO [wt%]	MgO [wt%]	Fe_2O_3 [wt%]	TiO_2 [wt%]	LOI	Mg#	Ni [ppm]	V/Sc	λ1	λ2
HLY0102 D011 - 19	WVZ*	82.60	-6.19	12.8	10.73	8.35	10.05	1.54	0.02	62.2	151	8.7	0.6	-29.7
HLY0102 D8 - 12	WVZ*	82.53	-6.16	12.8	11.04	8.41	10.30	1.52	-0.23	61.8	139	8.8	-0.7	-29.7
PS 66 217-16 (B)	WVZ*	82.86	-6.15	12.8	10.52	7.89	10.54	1.77	0.01	59.7	126	9.5	0.0	-27.9
PS 86 -019 (B)	WVZ*	82.53	-6.15	12.8	10.58	8.37	10.13	1.63	-0.37	62.1	151	9.2	0.4	-19.7
PS 86 - 67 (VG)	WVZ*	82.53	-6.15	12.8	10.87	8.23	10.55	1.59	-0.58	60.7	137	8.8	-0.7	-31.6
PS 86 - 044 (VG)	WVZ*	82.53	-6.15	12.8	10.44	8.18	10.24	1.65	-0.45	61.3	145	9.1	0.7	-29.1
PS 59 216 (B)	WVZ*	83.09	-6.10	12.8	11.26	8.25	10.33	1.50	-0.17	61.3	126	8.6	-0.9	-36.6
PS 59 216-11 (B)	WVZ*	83.09	-6.10	12.8	11.18	8.16	10.23	1.50	-0.18	61.2	129	8.8	-0.9	-34.8
PS 59 216-13 (B)	WVZ*	83.09	-6.10	12.8	11.34	8.34	10.38	1.51	-0.23	61.4	130	8.7	-0.6	-37.1
HLY0102 D013 - 10	WVZ*	83.20	-5.43	12.8	11.11	7.57	11.06	1.63	1.48	57.6	115	9.4	-1.5	-37.6
PS 59 231 - 14	WVZ*	83.96	-0.40	12.5	10.97	6.89	11.23	1.79	0.11	54.8	114	7.8	-3.8	-38.3
PS 59 232 - 84	WVZ*	84.17	0.88	12.3	10.81	8.32	10.37	1.53	-0.06	61.4	151	8.8	0.3	-31.4
HLY0102 D024 - 2	WVZ*	84.55	1.39	12.0	12.73	7.34	8.43	1.11	0.64	63.3	125	8.0	-1.8	-47.0
HLY0102 D026 - 9	WVZ*	84.28	2.34	12.3	10.94	8.15	10.91	1.70	-0.22	59.7	136	9.0	-1.0	-38.9
HLY0102 D018 - 1	WVZ*	83.42	2.54	12.7	10.96	7.70	10.77	1.71	-0.18	58.6	117	9.5	-0.7	-36.9
PS 59 234 - 24	WVZ*	84.57	3.13	12.0	10.88	9.92	10.12	1.46	-0.25	66.0	105	8.4	-3.6	-32.6
PS 59 224 - 34	WVZ*	83.38	3.66	12.7	10.49	6.50	10.28	1.74	1.71	55.6	77	10.0	1.3	-14.6
PS 59 243 - 36	SMZ*	84.97	10.15	11.7	11.19	7.74	8.98	1.19	0.30	63.0	155	7.2	0.6	-15.0
HLY0102 D037 - 5	SMZ*	85.18	11.30	11.7	9.39	6.89	10.82	1.79	-0.01	55.8	112	9.2	3.1	-4.6
HLY0102 D036 - 35	SMZ*	85.16	12.20	11.7	9.74	7.45	9.23	1.55	0.32	61.5	139	8.6	4.6	18.5
HLY0102 D038 - 6	SMZ*	85.19	12.42	11.7	10.45	8.70	9.71	1.42	-0.02	64.0	171	8.2	2.1	-1.7
PS 59 251 - 4	SMZ*	85.71	20.23	11.4	10.26	9.96	8.92	1.72	0.99	68.9	303	8.9	5.7	6.6
PS 59 263 - 26	EVZ	86.04	30.63	11.1	10.77	6.74	9.91	1.50	0.69	57.4	91	8.4	-3.1	-33.0
HLY0102 D050 - 30	EVZ	86.20	37.47	11.1	10.97	9.10	8.62	1.32	-0.01	67.6	198	7.8	3.4	-2.2

HLY0102 D051 - 15	EVZ	86.33	43.29	11.1	11.20	8.89	8.74	1.37	-0.21	66.8	170	7.9	2.3	-20.1
PS 101 186 R1 Basalt	EVZ	86.58	55.41	10.7	11.10	9.28	8.75	1.27	-0.05	67.7	191	10.2	0.7	-43.3
PS 101 186 VG	EVZ	86.58	55.41	10.7	11.01	9.33	8.95	1.29	-0.39	67.4	184	11.2	0.6	-33.9
PS 59 294 - 40	EVZ	86.90	56.41	10.7	11.13	9.33	9.35	1.32	0.20	66.4	63	8.2	-4.2	-51.1
PS 101 193 R1	EVZ	86.44	61.25	10.7	11.33	9.68	9.57	1.34	1.66	66.7	242	11.4	1.5	-25.8
PS 101 203 R4	EVZ	86.53	61.37	10.7	9.08	5.85	6.95	1.21	3.10	62.5	63	10.1	1.5	-31.5
PS 101 203 R5	EVZ	86.53	61.37	10.7	9.21	6.63	7.33	1.25	2.93	64.2	60	11.3	1.3	-31.2
PS 101 203 R8	EVZ	86.53	61.37	10.7	8.30	8.06	9.51	1.62	3.11	62.7	90	11.0	0.8	-35.0
PS 59 274 - 52	EVZ	86.73	66.76	10.7	10.78	9.36	9.39	0.98	-0.37	66.4	200	6.8	-0.2	-13.4
HLY0102 D059 - 35	EVZ	86.27	70.59	11.1	10.43	9.52	9.42	1.51	-0.15	66.7	224	8.8	2.7	-15.4
HLY0102 D061 - 11	EVZ	85.38	84.60	10.3	11.07	8.85	8.81	1.37	-0.26	66.6	180	8.4	1.7	-33.7

VG - Volcanic
Glass

λ0,1,2 after O'Neil (2016)

*- data from Richter et al. [53]

Supplementary Data Table S3 – Iron isotope data for rock reference materials BCR1 and BHVO1. n = Number of analyses. # Uncertainty is given as two standard error calculated from the number of analyses and the Student's *t* correcting factor.

Reference material	δ^{56}Fe [‰]	±2SE#	δ^{57}Fe [‰]	±2SE#	n
BCR1_1	+0.06	0.03	+0.07	0.04	3
BCR1_2	+0.03	0.02	+0.06	0.02	3
BCR1 _3	+0.08	0.03	+0.10	0.02	3
BCR1_rep	+0.12	0.01	+0.16	0.04	3
BCR1 average	***+0.07***	***0.02***	***+0.10***	***0.03***	***12***
BHVO1_1	+0.06	0.02	+0.12	0.03	4
BHVO1_2	+0.11	0.02	+0.18	0.02	3
BHVO1 _3	+0.14	0.03	+0.19	0.05	3
BHVO1_rep	+0.09	0.06	+0.11	0.04	2
BHVO1 average	***+0.10***	***0.02***	***+0.15***	***0.03***	***12***
